# Editorial for the Special Issue “Diabetes, Hypertension, and Cardiovascular Diseases: New Insights, Risk Factors, and Drug Therapies”

**DOI:** 10.3390/medicina62061128

**Published:** 2026-06-10

**Authors:** Panagiotis Theofilis, Emmanouil Mantzouranis, Christina Chrysohoou, Konstantinos Tsioufis

**Affiliations:** First Department of Cardiology, Hippokration General Hospital of Athens, National and Kapodistrian University of Athens, 115 27 Athens, Greece; panos.theofilis@hotmail.com (P.T.); mantzoup@gmail.com (E.M.)

Cardiovascular disease (CVD) remains the leading cause of morbidity and mortality worldwide [1], with diabetes and hypertension acting as major, interrelated drivers of this burden. Traditionally, these conditions have been approached as distinct clinical entities. However, accumulating evidence over the past decade has reshaped this perspective, highlighting the existence of a tightly interconnected cardio-renal-metabolic continuum which necessitates integrated approaches to risk stratification, prevention, and treatment [2]. This Special Issue brings together a diverse set of contributions that collectively reflect this shift. Rather than focusing on isolated disease processes, the included studies span biomarkers, interventional approaches, pharmacotherapy, and digital health, illustrating the multidimensional nature of cardiometabolic disease and its management.

A central theme emerging from this collection is the persistent challenge of residual cardiovascular risk despite advances in standard care. Bampali et al., through a systematic review and meta-analysis, demonstrated that the triglyceride-glucose (TyG) index is independently associated with an increased risk of major adverse cardiovascular events in patients with acute coronary syndromes (ACSs), reinforcing the role of insulin resistance as a clinically relevant determinant of outcomes even in the context of contemporary management (Contribution 1). Similarly, a systematic review on growth differentiation factor 15 (GDF-15) underscored its role as a biomarker of inflammatory and metabolic stress, linking obesity, subclinical atherosclerosis, and heart failure, and highlighting its potential to reflect shared pathophysiological pathways across the cardio-renal-metabolic continuum (Contribution 2). These contributions emphasize that traditional risk factors and scoring systems may be insufficient to fully capture individual risk profiles, pointing to the need for more nuanced and integrative biomarkers. Complementing these findings, real-world data from primary care settings demonstrate persistently suboptimal metabolic control among patients with diabetes, with only a minority achieving recommended glycemic targets and variable attainment of lipid and blood pressure goals, thereby underscoring the ongoing gap between guideline-directed care and everyday clinical practice (Contribution 3).

Beyond risk stratification, several studies in this Special Issue explore the interplay between systemic metabolic factors and cardiovascular interventions. The retrospective analysis of STEMI patients undergoing PCI identifies a high burden of in-stent restenosis in those selected for follow-up angiography and underscores the role of clinical comorbidities (diabetes, chronic kidney disease, and prior cardiovascular events) in shaping restenosis risk, while procedural characteristics appear less influential (Contribution 4). Regarding the prospective REFRAIN study, which evaluated distal renal denervation in patients with resistant hypertension and type 2 diabetes, it demonstrated preservation of renal function over 12 months despite significant blood pressure reduction, suggesting a potential nephroprotective effect of sympathetic modulation in this high-risk population (Contribution 5). The study on cardiac resynchronization therapy (CRT) further expands this perspective by highlighting its capacity to induce systemic and myocardial cardiometabolic adaptations, including improved substrate utilization, normalization of circulating energy metabolites, and favorable effects on ventricular function and ventriculo-arterial coupling (Contribution 6). Together, these works challenge the notion that interventional cardiology operates independently of systemic disease processes and underscore the importance of integrating metabolic context into procedural decision making.

Pharmacological innovation represents another major pillar of this Special Issue, particularly through the lens of sodium–glucose cotransporter 2 (SGLT2) inhibitors. The real-world study examining early initiation of SGLT2 inhibitors in ACS patients suggests that, despite a pronounced baseline risk profile characterized by lower left ventricular ejection fraction and greater hypertrophy, early therapy may be associated with stabilization of cardiac structure and a recovery trajectory comparable to that of lower-risk patients (Contribution 7). This is complemented by a comprehensive review that situates SGLT2 inhibitors within the evolving concept of cardio-renal-metabolic syndrome, emphasizing their multifaceted mechanisms of action and broad clinical benefits (Contribution 8). Together, these contributions reinforce the transition from glucose-centric management toward therapies that confer pleiotropic effects across the spectrum of cardiometabolic disease [3,4].

The inclusion of work on artificial intelligence (AI) and advanced digital health technologies in hypertension reflects the growing importance of data-driven approaches in modern cardiovascular care [5]. Recent advances highlight the potential of AI-enabled platforms, including remote blood pressure monitoring systems, machine learning-guided treatment titration, and digital twin models, to improve hypertension control and reduce therapeutic inertia [6]. Explainable AI has emerged as a critical component for fostering clinician trust and facilitating integration into clinical decision making [6]. However, significant challenges remain, including the need for prospective validation in real-world settings, seamless integration into clinical workflows, and robust frameworks addressing data quality, interpretability, privacy, and equity [7,8]. Emerging strategies such as federated learning and co-design approaches may enhance scalability and ensure broader applicability across diverse populations. The digital transformation of healthcare, while inevitable, must be carefully aligned with clinical needs and patient-centered outcomes.

Taken together, these contributions underscore both progress and persistent gaps in the field. Key challenges include the limited clinical integration and prospective validation of emerging biomarkers, insufficient consideration of metabolic phenotypes in interventional studies, uncertainties regarding the optimal timing and combination of novel therapies such as SGLT2 inhibitors [9], and the lack of outcome-driven evidence and scalable implementation strategies for AI and digital health tools. Addressing these gaps will require (1) the development of integrated risk models combining clinical, biomarker, and digital data, (2) phenotype-driven clinical trials, (3) deeper mechanistic insights into cardio-renal-metabolic interactions, and (4) stronger emphasis on implementation science to bridge the gap between evidence and practice.

## Data Availability

Not applicable.
